# A computational framework for predicting obesity risk based on optimizing and integrating genetic risk score and gene expression profiles

**DOI:** 10.1371/journal.pone.0197843

**Published:** 2018-05-24

**Authors:** Paule V. Joseph, Yupeng Wang, Nicolaas H. Fourie, Wendy A. Henderson

**Affiliations:** 1 Division of Intramural Research, National Institutes of Health, Bethesda, Maryland, United States of America; 2 Phronetik Inc., Plano, Texas, United States of America; GeneDx, UNITED STATES

## Abstract

Recent large-scale genome-wide association studies have identified tens of genetic loci robustly associated with Body Mass Index (BMI). Gene expression profiles were also found to be associated with BMI. However, accurate prediction of obesity risk utilizing genetic data remains challenging. In a cohort of 75 individuals, we integrated 27 BMI-associated SNPs and obesity-associated gene expression profiles. Genetic risk score was computed by adding BMI-increasing alleles. The genetic risk score was significantly correlated with BMI when an optimization algorithm was used that excluded some SNPs. Linear regression and support vector machine models were built to predict obesity risk using gene expression profiles and the genetic risk score. An adjusted R^2^ of 0.556 and accuracy of 76% was achieved for the linear regression and support vector machine models, respectively. In this paper, we report a new mathematical method to predict obesity genetic risk. We constructed obesity prediction models based on genetic information for a small cohort. Our computational framework serves as an example for using genetic information to predict obesity risk for specific cohorts.

## Introduction

Overweight and obesity, which are often indicated by high Body Mass Index (BMI), are growing significant health problems with significant public health and economic implications [1]. It is known that hereditary factors play a role in the development of obesity and increase the risk of many diseases such as cardiovascular disease and diabetes [2, 3]. In the clinical setting, risk assessment plays a pivotal role in the development of individualized prevention strategies and therapy for obesity and other associated metabolic diseases. In addition, recent recognition of obesity as a disease calls for change on how such complex issues are addressed by clinicians [4]. Therefore, it is important that efforts to personalize health in this area expand beyond assessment of the traditional risk factor categories (e.g., age, sex, physical activity) [5]. Use of a single gene variant to predict risk for diseases such as diabetes and obesity may be challenging because more than one gene may contribute toward the additive risk [6].

In the past five years, large genome wide association studies have identified novel genetic factors associated with obesity. Genome wide scans have generated data that help researchers better understand why some people are more predisposed to obesity than others. Thus, this project supports the application of translational genomics. To that end, the Genetic Investigation of Anthropometric Traits (GIANT) consortium has focused on identifying genetic loci that regulate human body size and shape, including height and measures of obesity, and have generated significant results [7]. A GWAS study conducted by this group identified 18 new loci with 32 Single Nucleotide Polymorphisms (SNPs) associated with obesity [8], and a more recent study identified an additional 11 new loci for anthropometric traits [9].

The availability of BMI-associated SNPs has enabled prediction of BMI or obesity based on genetic information. In addition to linear regression models using BMI-associated SNPs as variables, another common approach is to compute a genetic risk score [10], which sums up the number of BMI-increased alleles in any genome, and correlate the genetic risk score with obesity risks. However, existing prediction models do not achieve predictive accuracy high enough for clinical diagnosis or treatment decision making [11, 12].

Human genomes are complex, and ancestral differences in genetic variants may confound the effects of BMI-associated SNPs. For example, Zhu et al. concluded that BMI-associated SNPs tend to show lower effects in Han Chinese than in Europeans [12]. One reason for this observation maybe due to identified BMI-associated SNPs that may function differently in different ethnic groups. Such subtle differences have not been adequately investigated or quantitatively demonstrated. It may be necessary to assign different weights to these BMI-associated SNPs for generating an overall genetic risk score for obesity.

Functional genomic features such as gene expression profiling are also critical for understanding how genes perform biological functions that may further lead to diseases [13]. Recent studies suggest that some genes and biological pathways are associated with obesity risk [14, 15].Thus, it is possible that obesity risk could be more accurately predicted if BMI-associated SNPs are carefully selected to suit the structure of the investigated population and functional genomics features are included in the prediction models. In this study, we built predictive models for BMI by integrating the genotypes of BMI-associated SNPs and gene expression profiles.

## Materials and methods

### Ethics statement

This study was conducted in accordance with the Declaration of Helsinki of the World Medical Association. All study participants provided written informed consent. The research was reviewed and approved by the Institutional Review Board and the Office of Human Subjects Research at the National Institutes of Health (NIH). Written consent was obtained from adults. Children (ages 13–18) with the ability to read and understand assessment questionnaires provided assent in addition to parental consent.

### Design and setting

The clinical and genomics data of this study were originally obtained from 99 participants who were recruited under a natural history protocol (Clinicaltrial.gov #NCT00824941) conducted at the National Institutes of Health (NIH), Hatfield Clinical Research Center, in Bethesda, MD, USA from January 2009 to December 2015. Blood samples were collected from fasting participants during the first visit. BMI data were obtained for 90 participants. Baseline demographic characteristics are shown in Table 1.

**Table 1:** Baseline demographic characteristics of the 90 participants with BMI data.

Characteristic	Values
Sex, n (%)	
Male	44 (48.89)
Female	46 (51.11)
Age, y, mean (range)	28.16 (13─45)
BMI, mean (range)	26.21 (18.65─46.66)
Race, n (%)	
Asian	14 (15.56)
Black or African American	23 (25.56)
White	46 (51.11)
Other	7 (7.78)

### DNA extraction and genotyping OpenArray

Peripheral whole blood was collected from study participants and frozen immediately at -80°C until the time of extraction. DNA extraction was performed on 5 mL of blood using an Autopure instrument using Puregene reagents (Qiagen, Valencia, CA). DNA concentration was determined by NanoDrop™ 1000 spectrophotometer (ThermoScientific, Wilmington, DE) and extracted DNA was stored at -20°C prior to genotyping assay[16]. We genotyped 32 tagging SNPs of which 27 were BMI-associated SNPs from Speliotes et al.[8]using the Applied Biosystems TaqmanOpenArray genotyping platform following manufacture’s protocol (Life Technologies, Carlsbad, USA). Samples (n = 94) were genotyped in duplicate and samples with<80% call rate on the OpenArray platform were excluded. Genotypes were assigned using ABI's Genotyper software for OpenArrayTaqman data.

### RNA isolation, amplification

Blood samples (2.5 mL) from each participant were collected using PAXgene^TM^ RNA (Qiagen, Valencia, CA) tubes the morning after an overnight fast and frozen at -80°C. Total RNA was extracted and purified from each blood sample using an RNA PAXgene kit (Qiagen, Valencia, CA) according to the manufacturer's protocol and stored at-80°C. The RNA quantity, purity, and integrity were assessed via spectrometry and by using the RNA 6000 Nano LabChip kit on a 2100 Bioanalyzer (Agilent Technologies, Santa Clara, CA). Samples in which total RNA passed quality control criteria were used for microarray [17].

### Microarray data processing and annotation

Microarrays were processed by one technician at the Laboratory of Molecular Technology, National Cancer Institute (NIH, Frederick, MD), following standard protocol to minimize non-biologic technical bias. A total of 90 whole blood samples were assayed by Affymetrix GeneChip Human Genome U133 Plus 2.0 Array in two batches: 30 samples in the first batch and 60 samples in the second batch. Quality control (QC) was performed on the CEL files of each batch using array QualityMetrics. The software provided six QC metrics, including outlier detection by 1) distances between arrays, 2) boxplots, 3) relative log expression, 4) normalized unscaled standard error, 5) MA plots, and 6) spatial distribution of M. Any sample that failed three or more QC metrics was removed from analysis, resulting in a total of 84 samples with valid gene expression data.

Expression data within each batch were generated by the RMA approach [17], available in the Bioconductor “Affy” package. Then, microarray expression data from the two batches were merged and normalized using quantile normalization [16].Then, the Combat software [18] was implemented to remove the batch effects in the microarray expression data. To remove the effects of cross-hybridization the microarray expression data, we selected only the probe sets whose probes were mapped to a unique transcript of the UCSC known gene dataset [19]. We used BLASTN to map each probe sequence to the database of exons. Only perfect BLASTN hits (100% identify and 25 alignment length) were kept. We selected the probe sets if all of their probes were mapped to the same transcript. A list of 20,300 probe sets was generated. For the genes with multiple probe sets, we chose the probe set with the highest average expression level. A total of 13,276 gene expression profiles were analyzed in this study.

### Correction for multiple testing

Multiple testing was corrected using the Benjamin-Hochberg (BH) or False Discovery Rate (FDR) method [20].

### Additional quality control procedure

SNP data were carefully processed to account for missing genotypes. If a sample had 20% or more missing genotypes, it was removed from the analysis. Thus, a total of 87 samples were kept from the 94 participants. Out of the 87 samples, eight samples without BMI information were further removed. Out of the 79 samples, four samples did not have valid gene expression data. The total number of samples for integrating gene expression, SNP and BMI data was 75.

### Computing the genetic risk score

For each sample, a genetic risk score was computed. If the genotype of an SNP was homozygous for the "affect allele," a value of 2 was assigned for that SNP. If the genotype was heterozygous for the "affect allele/other allele," a value of 1 was assigned. If the genotype was homozygous for the "other allele," a value of 0 was assigned. The genetic risk score was the summation of the values of included SNPs divided by the number of included SNPs.

### Feature selection algorithm for SNP data

An optimization algorithm was designed to find the subset of SNPs whose genetic risk scores significantly correlated with BMI, based on the 81 samples with both SNP and BMI data. More specifically, the novel pipeline for the predictive modeling algorithm started with including all SNPs, and then implemented an iteration procedure. At each iteration, each SNP was assessed whether excluding this SNP would lead to higher correlation between BMI and the genetic risk score. The SNP with the highest correlation increase was chosen for exclusion at each iteration. The iteration procedure stopped when the correlation could not be increased.

### Feature selection algorithm for microarray data

An algorithm was designed to identify the gene expression profiles that were associated with BMI, based on the 84 samples with both gene expression and BMI data. First, each gene expression profile was assessed for its correlation with BMI and the corresponding *P*-value. Then, the *P*-values of all genes were adjusted for multiple testing, and the genes with adjusted *P*-value<0.5 were selected. However, not all the significant gene expression profiles were used because there may be dependency among these genes. To exclude the dependency among the significant genes, the following procedure was implemented:

1. The significant genes were ranked by the increasing of *P*-values.
2. An empty feature set was initiated.
3. The top associated (lowest *P*-value) gene was added to the feature set.
4. For the second to the last significant gene, it was assessed whether the gene could be added to the feature set. If the gene was not significantly correlated with any gene from the feature set, the gene was added to the feature set. Otherwise, it was excluded.

### Predictive modeling

Integrated prediction models for either the quantitative trait BMI or binary trait weight status were built based on the relevant features selected from microarray data and SNP data. We used multi-variable linear regression to predict BMI and support vector machine to predict weight status. Note that not all selected features from the microarray data were used to construct the integrated prediction model, as in the linear regression model, the fourth and fifth gene expression profiles were insignificant when all variables were included. The final linear regression model included six gene expression profiles and the genetic risk score from the selected SNPs.

$${BMI}_{i}={\alpha_{0}+\alpha }_{1}E_{i1}+\alpha_{2}E_{i2}+\alpha_{3}E_{i3}+\alpha_{4}E_{i4}+\alpha_{5}E_{i5+}\alpha_{6}E_{i6}+\beta G_{i}+\varepsilon_{i}$$

A prediction model for binary weight status was built using the support vector machine algorithm. The SVM software used was LibSVM [21]. The predictors were the same as the linear regression model. Different kernels were tested to generate the best performance. Five-fold cross-validation was used to ameliorate the overfitting problem.

## Results

### Study data

Whole-blood samples were assayed for gene expression using microarrays and genotyped for BMI-associated SNPs. Sample processing was previously described [16] and microarray data were batch corrected. A total of 27 BMI-associated SNPs from Speliotes et al. [8] were genotyped. Description of these SNPs is shown in S1 Table. After quality control was implemented (see Methods), a total of 84 samples were used to relate gene expression data to BMI (S1 Dataset), 79 samples were used to relate SNPs to BMI (S2 Dataset), while 75 samples (i.e. the overlap of the above two sets) were used to integrate gene expression, SNP and BMI data. The phenotype is either BMI (quantitative) or weight status (normal weight or overweight), determined by a BMI threshold of 25 for overweight).

### Validation analysis of microarray and SNP data by allelic-specific expression analysis

SNPs and gene expression are often related [22, 23].SNPs located in non-coding regions may affect the functionality or efficiency of *cis*-elements, and SNPs located in coding regions, especially in non-synonymous sites, may affect the functionality or structure of coded proteins. These relationships also provide an opportunity to validate whether SNP and microarray data for the same samples are reasonable and reflect biological phenomenon. To this end, we assessed potential allele-specific expression from the SNP and microarray data. For each SNP, we used the Analysis of Variance (ANOVA) model to examine whether the gene expression levels of its nearby gene were different depending on different genotypes of the SNP. After correcting for multiple testing, we detected five SNPs which rendered allelic specific expression (Table 2). All five SNPs are regulatory SNPs that are located either in intronic or intergenic regions. This analysis validates an integrative analysis of SNP and microarray data. We searched the GTEx database for possible eQTLs among the five SNP-gene pairs in the whole blood tissue and found a strong eQTL (*P* = 4.6x10^-9^, effect size = 0.32) for the fourth pair, i.e. rs2815752 and NEGR1.

**Table 2:** Identified SNP (genotype) and expression associations by ANOVA model.

SNP	Gene	Ensembl annotation	P-value	Adjusted P-value
rs13078807	CADM2	Intron variant	0.0126	0.077
rs10938397	GNPDA2	Intergenic variant	0.0243	0.0855
rs571312	MC4R	Intergenic variant	0.0252	0.0855
rs2815752	NEGR1	Intergenic variant	0.00373	0.0634
rs4929949	RPL27A	Intron variant	0.0136	0.0772

### Selection of BMI-associated SNPs using an optimization procedure

Genetic risk score for obesity was computed by adding BMI-increasing alleles. We computed the correlation between the genetic risk score of 27 BMI-associated SNPs and BMI for the entire investigated cohort but found the correlation coefficient was not significant (Pearson’s *r* = 0.089, *P* = 0.436; or Spearman’s *r* = 0.082, *P* = 0.473). However, because the BMI-associated SNPs were found to have different effect sizes in different populations, it is possible that not all the pre-selected BMI-associated SNPs may have actual effects for a cohort with specific population structure. Thus, we designed a feature selection procedure that excludes BMI-associated SNPs one by one to achieve an optimal correlation between genetic risk score and BMI, determined by the convergence of the correlation. We examined the effectiveness of this procedure by applying it to the cohort of 81 samples. The correlations corresponding to different numbers of excluded SNPs were listed (Table 3). Based on Spearman’s correlation, the correlation coefficient reached convergence when 18 SNPs were excluded, while this number was 16 using Pearson’s correlation. The optimal correlation coefficients were 0.527 (*P* = 6.0×10^−7^) and 0.478 (*P* = 8.5×10^−6^) for Spearman and Pearson’s correlations, respectively, with both being statistically significant. This analysis suggests that the correlation can be optimized when some SNPs are excluded, also indicating that the genetic risk score may be significantly correlated with BMI when proper SNPs are selected.

**Table 3:** Correlations between genetic risk score and BMI-associated SNPs.

Number of excluded SNPs	Pearson		Spearman	
	P	r	P	r
1	0.248	0.131	0.250	0.131
2	0.103	0.185	0.113	0.180
3	0.050	0.221	0.058	0.215
4	0.023	0.255	0.017	0.269
5	0.011	0.285	7.8×10^−3^	0.297
6	4.8×10^−3^	0.314	1.4×10^−3^	0.354
7	2.0×10^−3^	0.343	5.6×10^−4^	0.380
8	8.4×10^−4^	0.368	1.0×10^−4^	0.423
9	3.5×10^−4^	0.393	5.3×10^−5^	0.438
10	1.7×10^−4^	0.410	2.7×10^−5^	0.453
11	6.9×10^−5^	0.432	1.5×10^−5^	0.466
12	2.8×10^−5^	0.453	7.6×10^−6^	0.480
13	1.1×10^−5^	0.473	6.3×10^−6^	0.484
14	9.9×10^−6^	0.475	6.0×10^−6^	0.485
15	9.3×10^−6^	0.476	5.1×10^−6^	0.488
16	**8.5×10**^**−6**^	**0.478**	1.4×10^−6^	0.512
17	8.7×10^−6^	0.477	1.2×10^−6^	0.515
18	7.0×10^−6^	0.482	**6.0×10**^**−7**^	**0.527**
19	7.6×10^−6^	0.480	6.0×10^−7^	0.527
20	1.1×10^−5^	0.472	8.8×10^−7^	0.521

### Predictive modeling for obesity risks

Gene expression levels can be another layer of genetic information for predicting obesity risks. We designed a feature selection procedure to select several uncorrelated gene expression profiles from BMI-associated gene expression profiles (see Methods). Expression profiles were significantly correlated with BMI using Pearson’s correlation coefficient. However, 52 expression profiles were found to be correlated with BMI using Spearman’s correlation coefficient. A total of six independent expression profiles including *ADPGK*, *RIOK3*, *CEP41*, *ZFP57*, *HOXA3* and *CXorf27*, and three independent expression profiles including *ICAM4*, *SLC30A3*and *HERPUD1* were selected for Pearson’s and Spearman’s correlation coefficients respectively, using the feature selection algorithm described in the Methods. Next, we built linear regression models for BMI using expression profiles for the cohort of 77 samples with gene expression, SNP and BMI data. We found that the coefficients of the six genes chosen according to Pearson’s correlation coefficient were significant, resulting in a maximum adjusted R^2^ of 0.527 (*P* = 1.0×10^−10^) when all of the six genes were included. In contrast, using Spearman’s correlation coefficient, the maximum adjusted R^2^was only 0.291 when all of the three genes were included. Thus, we decided to use the genes and SNPs chosen based on Pearson’s correlation coefficient for subsequent analysis. Then, we examined linear regression models involving both genetic risk score and gene expression profiles. As described above, a total of 11 SNPs were selected (16 were excluded). These SNPs includedrs7359397, rs9816226, rs29941, rs543874, rs987237, rs713586, rs1514175, rs887912, rs13078807, rs1555543 and rs206936. The coefficient of genetic risk score was significant (*P* = 2.3×10^−2^), and the linear regression models generated an adjusted R^2^ of 0.556 (*P* = 3.5×10^−11^), which is higher than the R^2^ (0.527) obtained without inclusion of the genetic risk score. Detailed variable selection procedure and parameter estimates are shown in S1 File. This analysis indicates that the genetic risk score can significantly improve the obesity prediction model when the SNPs for computing the score are properly selected. Further, we asked whether potential interaction effects among gene expression profiles and genetic risk score could improve explanation of BMI. We added an interaction term in the linear regression model of BMI and assessed all pair-wise combinations among the six gene expression profiles and genetic risk score. However, after adjusting for multiple testing, none interaction term was statistically significant (S1 File). This analysis suggests that the primary effects among the six gene expression profiles and genetic risk score are additive effects. The data for the final linear regression model are deposited in S3 dataset.

Then, a machine learning model based on SVM was built for the binary weight status using the aforementioned six gene expression profiles and the genetic risk score. Using five-fold cross-validation and the linear SVM, the model yielded an accuracy of 76%. This accuracy is moderate, which is consistent with the moderate adjusted R^2^ of the linear regression model. The concordance between the two approaches suggests that our predictive modeling for obesity risks is robust. Our predictive modeling improved the model and suggests that feature selection of genetic markers may be necessary for building practical prediction models for obesity risk. Then, using ToppFun function in ToppGene Suite https://toppgene.cchmc.org [24] to generate a gene list functional enrichment analysis to find links of our results to prior studies in the literature, phenotype and disease (see, Table 4). We found that the top human phenotypes were obesity, increase adipose tissue, abnormal energy expenditure, abnormal homeostasis, acanthosis nigricans, polyphagia, and eating disorders. The gene-disease associations were primarily from previously reported Genome-wide Association Studies (GWAS) and the Database of Human disease-associated genes and variants (DisGeNET).

**Table 4:** Multiomic/Phenotype concordance of the 5 SNPs.

Reference	Associated Phenotype	Genes from input
Speliotes et al. 2010 [8]	Body Mass Index	MC4R CAMD2 GNPDA2 NEGR1 RPL27A
Berndt et al. 2013 [9]	Anthropometric traits	MC4R CAMD2 GNPDA2 NEGR1 RPL27A
Orkunoglu-Suer et al. 2011 [25]	Body Mass Index	MC4R GNPDA2 NEGR1
Elks et al. 2010 [26]	Weight gain and growth	MC4R GNPDA2 NEGR1
Renstrom et al. 2009 [27]	Obesity	MC4R GNPDA2 NEGR1

## Discussion

**Fig 1:**
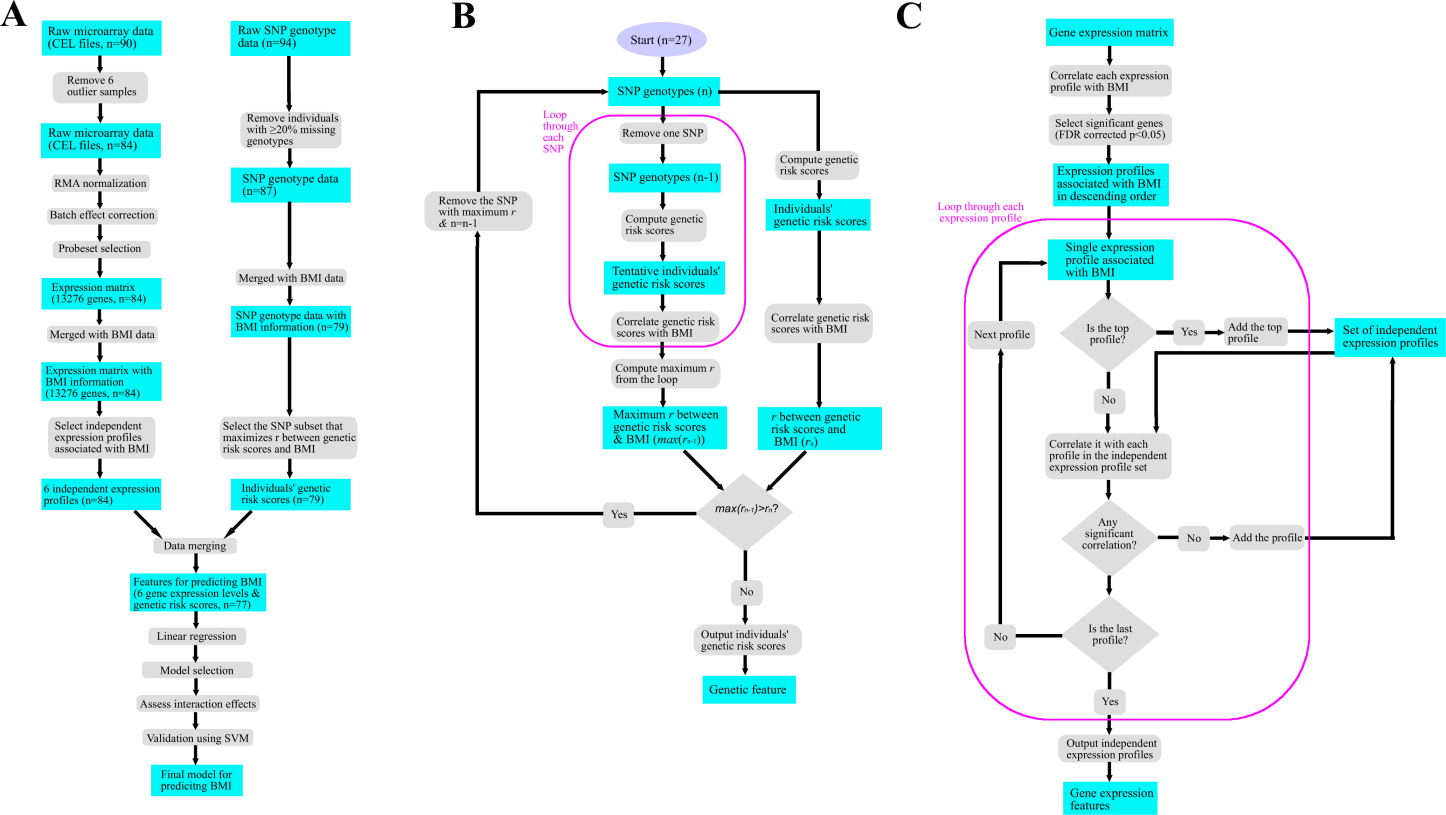
Flow charts for the computational procedures. A) Flow chart of the entire procedure of data processing and analysis. B) Flow chart of the feature selection algorithm for SNP data. C) Flow chart of the feature selection algorithm for microarray data.

There are different approaches to assess genetic associations between SNPs and quantitative traits. Individual SNPs were also assessed whether their genotypes were associated with BMI. The genotypes were coded by 0 (homozygous for the other allele), 1 (heterozygous), and 2 (homozygous for the affect allele). Three approaches for association testing were applied: correlation, linear regression, and ANOVA. Fig 1 depicts the algorithms used for gene correlation, SNP selection and the genetic risk scoring process. The code used for generating the genetic risk scores can be found in https://github.com/wyp1125/compute-grs. After adjustment for multiple testing, none of these methods yielded significant associations. In addition, individual SNPs were also assessed for Quantitative Trait Locus (QTLs) for BMI, using the R/qtl package [28]. Both single-locus and interval mapping based on expectation–maximization, Haley-Knott, and interference message passing algorithms were assessed. However, no QTL was detected. These results suggest that these obesity-associated SNPs are functional in an additive mode, which rationalizes the usage of the genetic risk score. As shown in Table 4 our results were validated externally with prior studies. All 5 genes generated by allelic-specific expression analysis has been associated with weight-related disorders. Notably, NEGR1 and MC4R are—found to be associated with obesity across multiple lines of evidence. This suggests a potential mechanistic basis for the regulation of those SNPs that may affect gene expression.

## Supporting information

S1 Table BMI-associated SNPs genotyped in this study. (DOCX)

S1 Dataset Combination of gene expression data with BMI information. (XLSX)

S2 Dataset Combination of SNP data with BMI information. (XLSX)

S3 Dataset Data for building the final linear regression model of BMI. (XLSX)

S1 File Variable selection and parameter estimates for linear regression models of BMI. (DOCX)
