# Supplementary material for: A computational framework for predicting obesity risk based on optimizing and integrating genetic risk score and gene expression profiles
Source: PLoS One. 2018 May 24;13(5):e0197843. doi: 10.1371/journal.pone.0197843 (PMC5993110; doi:10.1371/journal.pone.0197843)
Supplement: S1 Table — (DOCX) [file pone.0197843.s001.docx]

**S1 Table. BMI-associated SNPs genotyped in this study**

| **Other allele** | **Effective allele** | **Gene** | **SNP ID** |
| --- | --- | --- | --- |
| T | A | FTO | rs1558902 |
| C | A | MC4R | rs571312 |
| A | G | GNPDA2 | rs10938397 |
| T | A | BDNF | rs10767664 |
| G | A | NEGR1 | rs2815752 |
| C | T | SH2B1 | rs7359397 |
| A | T | ETV5 | rs9816226 |
| A | G | KCTD15 | rs29941 |
| A | G | SEC16B | rs543874 |
| A | G | TFAP2B | rs987237 |
| G | A | FAIM2 | rs7138803 |
| T | C | NRXN3 | rs10150332 |
| T | C | RBJ | rs713586 |
| T | C | GPRC5B | rs12444979 |
| A | G | MAP2K5 | rs2241423 |
| T | C | QPCTL | rs2287019 |
| G | A | TNNI3K | rs1514175 |
| G | T | FLJ35779 | rs2112347 |
| A | G | LRRN6C | rs10968576 |
| G | A | TMEM160 | rs3810291 |
| C | T | FANCL | rs887912 |
| A | G | CADM2 | rs13078807 |
| T | C | LRP1B | rs2890652 |
| A | C | PTBP2 | rs1555543 |
| A | G | MTIF3 | rs4771122 |
| T | C | RPL27A | rs4929949 |
| A | G | NUDT3 | rs206936 |
