# Supplementary material for: A computational framework for predicting obesity risk based on optimizing and integrating genetic risk score and gene expression profiles
Source: PLoS One. 2018 May 24;13(5):e0197843. doi: 10.1371/journal.pone.0197843 (PMC5993110; doi:10.1371/journal.pone.0197843)
Supplement: S1 File — (DOCX) [file pone.0197843.s005.docx]

**Variable selection and parameter estimates for linear regression models of BMI**

Models 1~6 assess the gene expression profiles that should be included in the final model. When all of the top six genes are included (model 5), all coefficients are significant, and the adjusted R^2^ is the highest.

1. ${BMI}_{i}={\alpha_{0}+\alpha}_{1}\cdot E_{i1}+\varepsilon_{i}$

| **Model summary** | | | | | |
| --- | --- | --- | --- | --- | --- |
| Adjusted R^2^: 0.1753 | | | Residual std error: 5.648 | | |
| F-statistic: 16.73 | | | *p*-value: 0.0001097 | | |
| **Coefficients** | | | | | |
| Term | Estimate | Std error | | *t* value | Pr(>\|*t*\|) |
| Intercept | 203.58 | 43.32 | | 4.70 | 1.2e-05 *** |
| $E_{1}$ | -15.38 | 3.76 | | -4.09 | 0.00011 *** |

2. ${BMI}_{i}={\alpha_{0}+\alpha}_{1}\cdot E_{i1}+\alpha_{2}\cdot E_{i2}++\varepsilon_{i}$

| **Model summary** | | | | | |
| --- | --- | --- | --- | --- | --- |
| Adjusted R^2^: 0.2981 | | | Residual std error: 5.211 | | |
| F-statistic: 16.71 | | | *p*-value: 1.09e-06 | | |
| **Coefficients** | | | | | |
| Term | Estimate | Std error | | *t* value | Pr(>\|*t*\|) |
| Intercept | 119.833 | 45.896 | | 2.611 | 0.010979 * |
| $E_{1}$ | -13.062 | 3.525 | | -3.706 | 0.000411 *** |
| $E_{2}$ | 5.453 | 1.469 | | 3.711 | 0.000404 *** |

3. ${BMI}_{i}={\alpha_{0}+\alpha}_{1}\cdot E_{i1}+\alpha_{2}\cdot E_{i2}+\alpha_{3}\cdot E_{i3}+\varepsilon_{i}$

| **Model summary** | | | | | |
| --- | --- | --- | --- | --- | --- |
| Adjusted R^2^: 0.3614 | | | Residual std error: 4.97 | | |
| F-statistic: 14.96 | | | *p*-value: 1.208e-07 | | |
| **Coefficients** | | | | | |
| Term | Estimate | Std error | | *t* value | Pr(>\|*t*\|) |
| Intercept | 152.799 | 45.276 | | 3.375 | 0.001200 ** |
| $E_{1}$ | -11.364 | 3.414 | | -3.328 | 0.001388 ** |
| $E_{2}$ | 4.904 | 1.415 | | 3.467 | 0.000899 *** |
| $E_{3}$ | -5.646 | 1.979 | | -2.853 | 0.005666 ** |

4. ${BMI}_{i}={\alpha_{0}+\alpha}_{1}E_{i1}+\alpha_{2}E_{i2}+\alpha_{3}E_{i3}+\alpha_{4}E_{i4}+\varepsilon_{i}$

| **Model summary** | | | | | |
| --- | --- | --- | --- | --- | --- |
| Adjusted R^2^: 0.4347 | | | Residual std error: 4.676 | | |
| F-statistic: 15.22 | | | *p*-value: 5.291e-09 | | |
| **Coefficients** | | | | | |
| Term | Estimate | Std error | | *t* value | Pr(>\|*t*\|) |
| Intercept | 164.1942 | 42.7493 | | 3.841 | 0.000266 *** |
| $E_{1}$ | -11.3398 | 3.2127 | | -3.530 | 0.000740 *** |
| $E_{2}$ | 4.2454 | 1.3469 | | 3.152 | 0.002388 ** |
| $E_{3}$ | -5.2411 | 1.8662 | | -2.808 | 0.006446 ** |
| $E_{4}$ | -1.3645 | 0.4273 | | -3.193 | 0.002108 ** |

5. ${BMI}_{i}={\alpha_{0}+\alpha}_{1}E_{i1}+\alpha_{2}E_{i2}+\alpha_{3}E_{i3}+\alpha_{4}E_{i4}+\alpha_{5}E_{i5}+\varepsilon_{i}$

| **Model summary** | | | | | |
| --- | --- | --- | --- | --- | --- |
| Adjusted R^2^: 0.4862 | | | Residual std error: 4.458 | | |
| F-statistic: 15 | | | *p*-value: 5.928e-10 | | |
| **Coefficients** | | | | | |
| Term | Estimate | Std error | | *t* value | Pr(>\|*t*\|) |
| Intercept | 167.1407 | 40.7685 | | 4.100 | 0.000111 *** |
| $E_{1}$ | -9.5775 | 3.1255 | | -3.064 | 0.003111 ** |
| $E_{2}$ | 3.8318 | 1.2924 | | 2.965 | 0.004154 ** |
| $E_{3}$ | -5.9686 | 1.7976 | | -3.320 | 0.001439 ** |
| $E_{4}$ | -1.1634 | 0.4135 | | -2.814 | 0.006374 ** |
| $E_{5}$ | -2.1995 | 0.7768 | | -2.832 | 0.006065 ** |

6. ${BMI}_{i}={\alpha_{0}+\alpha}_{1}E_{i1}+\alpha_{2}E_{i2}+\alpha_{3}E_{i3}+\alpha_{4}E_{i4}+\alpha_{5}E_{i5}+{\alpha_{6}E_{i6}+\varepsilon}_{i}$

| **Model summary** | | | | | |
| --- | --- | --- | --- | --- | --- |
| Adjusted R^2^: 0.5274 | | | Residual std error: 4.276 | | |
| F-statistic: 14.76 | | | *p*-value: 1.019e-10 | | |
| **Coefficients** | | | | | |
| Term | Estimate | Std error | | *t* value | Pr(>\|*t*\|) |
| Intercept | 104.0181 | 45.7899 | | 2.272 | 0.02628 * |
| $E_{1}$ | -8.6952 | 3.0160 | | -2.883 | 0.00527 ** |
| $E_{2}$ | 4.1391 | 1.2449 | | 3.325 | 0.00143 ** |
| $E_{3}$ | -5.1209 | 1.7535 | | -2.920 | 0.00474 ** |
| $E_{4}$ | -0.9135 | 0.4076 | | -2.241 | 0.02830 * |
| $E_{5}$ | -2.3326 | 0.7467 | | -3.124 | 0.00262 ** |
| $E_{5}$ | 8.2415 | 3.1115 | | 2.649 | 0.01004 * |

Model 7 assesses whether including genetic risk scores can improve the linear regression model of BMI, based on 11 SNPs selected according to Pearson’s correlation.

7. ${BMI}_{i}={\alpha_{0}+\alpha}_{1}E_{i1}+\alpha_{2}E_{i2}+\alpha_{3}E_{i3}++\alpha_{4}E_{i4}+\alpha_{5}E_{i5}+\alpha_{6}E_{i6}+\beta G_{i}+\varepsilon_{i}$

| **Model summary** | | | | | |
| --- | --- | --- | --- | --- | --- |
| Adjusted R^2^: 0.5563 | | | Residual std error: 4.143 | | |
| F-statistic: 14.26 | | | *p*-value: 3.468e-11 | | |
| **Coefficients** | | | | | |
| Term | Estimate | Std error | | *t* value | Pr(>\|*t*\|) |
| Intercept | 104.3655 | 44.3654 | | 2.352 | 0.02160 * |
| $E_{1}$ | -8.2618 | 2.9280 | | -2.822 | 0.00628 ** |
| $E_{2}$ | 3.4493 | 1.2419 | | 2.777 | 0.00710 ** |
| $E_{3}$ | -4.8627 | 1.7025 | | -2.856 | 0.00570 ** |
| $E_{4}$ | -0.8613 | 0.3956 | | -2.177 | 0.03298 * |
| $E_{5}$ | -1.8419 | 0.7534 | | -2.445 | 0.01713 * |
| $E_{6}$ | 6.3904 | 3.1174 | | 2.050 | 0.04429 * |
| $G$ | 8.0614 | 3.4570 | | 2.332 | 0.02272 * |

**Assessing pairwise interaction terms among gene expression profiles and genetic risk score**

Each pairwise interaction term was added to model 7 and the p-value of its coefficient was recorded. Then all the p-values were adjusted by the FDR method.

| **Interaction term** | **P-value** | **Adjusted p-value** |
| --- | --- | --- |
| $E_{1}E_{2}$ | 0.173349164 | 0.51191421 |
| $E_{1}E_{3}$ | 0.396789672 | 0.59518451 |
| $E_{1}E_{4}$ | 0.004226964 | 0.08876624 |
| $E_{1}E_{5}$ | 0.915778712 | 0.91577871 |
| $E_{1}E_{6}$ | 0.026222106 | 0.18355474 |
| $E_{2}E_{3}$ | 0.209795458 | 0.51191421 |
| $E_{2}E_{4}$ | 0.262638109 | 0.51191421 |
| $E_{2}E_{5}$ | 0.434626764 | 0.60847747 |
| $E_{2}E_{6}$ | 0.268145541 | 0.51191421 |
| $E_{3}E_{4}$ | 0.603791678 | 0.66734870 |
| $E_{3}E_{5}$ | 0.602067062 | 0.66734870 |
| $E_{3}E_{6}$ | 0.827632162 | 0.86901377 |
| $E_{4}E_{5}$ | 0.347625924 | 0.59518451 |
| $E_{4}E_{6}$ | 0.082691229 | 0.37646072 |
| $E_{5}E_{6}$ | 0.264693050 | 0.51191421 |
| $E_{1}G$ | 0.472277197 | 0.61986382 |
| $E_{2}G$ | 0.544045624 | 0.66734870 |
| $E_{3}G$ | 0.089633505 | 0.37646072 |
| $E_{4}G$ | 0.020313594 | 0.18355474 |
| $E_{5}G$ | 0.375587084 | 0.59518451 |
| $E_{6}G$ | 0.133763962 | 0.46817387 |
